# Tumor Mutational Burden as a Prognostic Biomarker in Follicular Lymphoma

**DOI:** 10.3390/cancers18050737

**Published:** 2026-02-25

**Authors:** Marta Lafuente, Ramón Diez-Feijóo, Marta García-Recio, Nieves Garcia-Gisbert, Maria Concepción Fernández-Rodríguez, Raquel Longarón, Junjie Ji, Bárbara Tazon-Vega, Sergio Pinzón, Lierni Fernández-Ibarrondo, Blanca Sánchez-González, Laura Camacho, Joan Gibert, Antonio Gutierrez, Beatriz Bellosillo, Antonio Salar

**Affiliations:** 1 Group of Applied Clinical Research in Hematology, Cancer Research Program Hospital del Mar Research Institute, 08003 Barcelona, Spain; 2 Department of Medicine and Life Sciences, Pompeu Fabra University, 08003 Barcelona, Spain; 3 Department of Hematology, Hospital del Mar, 08003 Barcelona, Spain; 4 Department of Hematology, University Hospital Son Espases/Health Research Institute of the Balearic Islands (IdISBa), 07120 Palma, Spain; 5 Department of Pathology, Hospital del Mar, 08003 Barcelona, Spain

**Keywords:** follicular lymphoma, tumor mutational burden, prognostic biomarker, t(14;18) translocation, next generation sequencing

## Abstract

Introduction: Follicular lymphoma (FL) is the most common indolent non-Hodgkin lymphoma. Approximately 20% of patients experience early relapse within 24 months (POD24) of immunochemotherapy (ICT), a subgroup associated with poor prognosis and not well-identified by current prognostic indices. Given that tumor mutational burden (TMB) has been associated with outcome in various cancers, we investigated its potential as a prognostic biomarker in FL. Methods: TMB was estimated by next-generation sequencing using a 409-gene panel in a cohort of 119 patients diagnosed with FL grades 1–3A and treated with frontline immunotherapy or ICT. Results: Although TMB was not associated with clinical variables in non-transformed FL patients, higher TMB values were observed in patients harboring the t(14;18) translocation, mutations in *BCL2*, *TNFRSF14*, or genes involved in cellular migration (*GNA13*, *GNAI2*). Notably, patients with high TMB (>2.55 mutations per megabase) showed longer progression-free survival, lymphoma-specific survival, and overall survival, and had fewer mutations affecting the mTORC1 pathway. A higher proportion of POD24 patients was observed in the low TMB subgroup. In 25 paired diagnosis-relapse samples, TMB remained globally stable, although variations in the molecular landscape were observed. In addition, we analyzed TMB in a separate cohort of patients presenting transformed FL at diagnosis (*n* = 16). Higher TMB was also associated with the t(14;18) chromosomal translocation, but not with clinical features or survival. Conclusions: These findings support the potential of TMB as a prognostic biomarker in FL, providing molecular understanding beyond established clinical indices and aiding in the identification of patients at higher risk of early progression following ICT.

## Simple Summary

Follicular lymphoma is a slow-growing type of blood cancer. Around 20% of patients do not respond or relapse early after standard first-line treatment and have a worse outcome. Currently, there are no clinical tools that accurately identify these high-risk patients at diagnosis. In this study, we explored whether the number of genetic alterations present in the tumor, known as tumor mutational burden, could help predict patient prognosis. We analyzed tumor samples obtained from patients with follicular lymphoma using next-generation sequencing and explored the relationship between genetic findings and clinical outcomes. Patients with more tumor mutations were less likely to experience early relapses and lived longer, while those with fewer mutations were more prone to relapse early. These results suggest that measuring tumor mutational burden may help improve risk stratification in follicular lymphoma.

## 1. Introduction

Follicular lymphoma (FL) represents the most common indolent subtype and the second most frequent overall among non-Hodgkin lymphomas in Western countries, with most patients experiencing an indolent course [1]. However, approximately 20–30% of patients relapse or progress within 24 months of front-line immunochemotherapy (POD24), leading to a decreased overall survival (OS) [2]. Moreover, around 1% of FL patients per year undergo histological transformation to a clinically aggressive lymphoma, typically diffuse large B-cell lymphoma (DLBCL) [3,4]. Prognostic indices based on clinical parameters have been developed to identify high-risk patients at diagnosis, including the Follicular Lymphoma International Prognostic Index (FLIPI) [5], the FLIPI-2 [6], or the PRIMA-PI [7]. However, these systems have limitations in predicting the disease course and treatment response in individual patients.

Recent efforts to improve FL prognostic indices include the incorporation of genetic data. While the t(14;18)(q32;q21) translocation is a hallmark of FL, additional genetic alterations contribute to disease progression [8,9,10]. This has led to the development of clinical genetic models such as m7-FLIPI [11] and POD24-PI [12], although FLIPI remains the most discriminative tool [13]. Nevertheless, the development of improved biomarkers is still needed to refine risk assessment and guide treatment decisions.

One promising biomarker in the field of oncology is tumor mutational burden (TMB), which is defined as the number of somatic mutations per megabase of DNA (mut/Mb) in a tumor’s genome. Tumors with high TMB are more likely to produce neoantigens that are recognized by the immune system and are more prone to trigger an anti-tumor immune response. TMB was initially recognized as a potential predictive biomarker for immune checkpoint inhibitor (ICI) therapy response [14,15]. More recently, it has also been proven to be a potential prognostic biomarker in the absence of ICI treatment in several types of cancer [16,17]. However, the prognostic value of TMB in hematological malignancies remains largely unexplored.

Although whole exome sequencing is considered the gold standard method for TMB assessment, its applicability in routine diagnostics remains limited due to the cost and turnaround time. TMB estimated using targeted next-generation sequencing (NGS) panels covering more than 1 Mb of coding regions has demonstrated a strong correlation with WES TMB, and has therefore been proposed as a more affordable alternative with shorter turnaround times [18,19,20].

In this study, we evaluated TMB through a 409-gene commercial panel in diagnostic samples from 119 de novo FL patients and 16 patients with de novo transformed FL (tFL). We analyzed the association of TMB with prognosis and specific genetic alterations recurrent in FL, with the aim of identifying molecular markers that could improve prognostic assessment.

## 2. Patients and Methods

### 2.1. Patients

Formalin-fixed paraffin-embedded (FFPE) tumor biopsies obtained at diagnosis were retrospectively collected from 135 treatment-naïve patients: 119 patients diagnosed with de novo FL grades 1–3A between 2002 and 2022, and 16 patients diagnosed with tFL between 2015 and 2022. All cases were diagnosed by expert hematopathologists at each center according to the WHO criteria, and the presence of the t(14;18)(q32;q21) translocation was evaluated by fluorescence in situ hybridization (FISH) on diagnostic tumor samples as part of clinical routine. Patients included in the study had received standard front-line treatment: rituximab (R) monotherapy, R or obinutuzumab (O) (anti-CD20) combined with CHOP or CHOP-like regimens, R-CVP, or R/O-Bendamustine (R/O-B). All patients who achieved a complete response (CR) or partial response (PR) started 2-year maintenance with R. Definitions of complete response (CR), partial response (PR), and progressive disease (PD) followed standard criteria [21]. Additionally, we assessed TMB evolution over time by analyzing FFPE tumor biopsies collected at the time of relapse from 25 of the 119 de novo FL patients. The study was conducted in accordance with the principles of the modified Declaration of Helsinki and approved by the ethics committees of both participating centers. All patients provided written consent for the use of remnant clinical samples for research purposes.

### 2.2. Clinical Endpoints

Response and progression were assessed by treating clinicians according to the Lugano Classification criteria [22]. Imaging assessments, including computed tomography (CT) and positron emission tomography (PET)/CT, as well as bone marrow evaluation, were performed at each center according to routine clinical practice. POD24 was defined as progression or relapse of the disease within the first 24 months after first-line treatment initiation (modified definition).

Progression-free survival (PFS), lymphoma-specific survival (LSS), and OS were calculated from the date of first treatment to the date of the event. Patients were censored at the last follow-up if the event did not occur. For PFS, the event was either progression or relapse of the lymphoma, or death from any cause; for LSS, it was death due to lymphoma; and for OS, death from any cause. Lymphoma-specific death was defined as death directly attributable to lymphoma progression. Treatment-related complications, such as fatal infections, cardiac events, or secondary malignancies, were not considered lymphoma-specific unless they occurred in the context of unequivocal lymphoma progression. Deaths clearly unrelated to lymphoma were censored at the time of death.

### 2.3. DNA Isolation

DNA extraction was performed with the QIAamp DNA Mini Kit (QIAGEN, Hilden, Germany) using the QIAcube system. To ensure a high-quality DNA input and reduce potential deamination artifacts, all DNA samples used were extracted shortly after FFPE processing. DNA concentration was measured using a Qubit Fluorometer (Thermo Fisher Scientific, Waltham, MA, USA).

### 2.4. Next Generation Sequencing (NGS)

TMB was evaluated using the Oncomine Tumor Mutation Load Assay (ThermoFisher Scientific), a targeted NGS assay that covers 1.65 Mb across 409 genes (1.2 Mb of exonic regions). NGS amplicon libraries were prepared from FFPE-extracted DNAs using Ion AmpliSeq Library Kit Plus (Thermo Fisher Scientific). The workflow requires 20 ng of input DNA at 2.67 ng/μL and generates 50 μL of the final library. Sequencing was carried out on the Ion GeneStudio S5 System (Thermo Fisher Scientific) with a 300× minimum mean read depth. Run performance was analyzed in Torrent Suite Software version 5.18 (Thermo Fisher Scientific), and NGS results were analyzed using Ion Torrent Software version 5.18 (Thermo Fisher Scientific). All samples showed a cytosine deamination score ≤1. The Oncomine Tumor Mutation Load w3.3 predefined workflow was slightly modified to obtain TMB estimates. After variant calling, germline variants were removed using population databases: Exome Aggregation Consortium, UCSC Common SNPs, 5000 Exomes, and 1000 Genome Project. The predefined workflow considers non-synonymous single-nucleotide variants (SNVs) and insertions/deletions located in exonic regions, with a variant allele frequency (VAF) ≥5%, and a read depth ≥60 reads. Moreover, variants located in homopolymeric regions longer than 4 bp were discarded. We included one additional filtration step to discard variants with less than 10 reads of the alternate allele. TMB was calculated by dividing the total number of variants by the total number of exonic bases with ≥60 reads.

### 2.5. Additional Genetic Data

For 64 out of the 119 de novo FL patients, information on the mutational status of 64 FL-relevant genes was available, including the 7 genes incorporated in the m7-FLIPI prognostic index. This enabled us to define the mutational profile and calculate the m7-FLIPI score in this subgroup of patients only, as these 7 genes were not all included in the 409-gene panel used for TMB estimation. Genes included in the 64-gene FL panel were categorized according to their biological function into six distinct biological pathways: cell survival (*ATM*, *BCL2*, *CCND3*, *DTX1*, *EEF1A1*, *FAS*, *JAK2*, *JAK3*, *MCL1*, *MYC*, *NOTCH1*, *NOTCH2*, *PIM1*, *SGK1*, *SOCS1*, *ST6GAL1*, *STAT6*, *TNIK*, *TP53*), epigenetic and transcriptional regulation (*ARID1A*, *ARID1B*, *BCL7A*, *CREBBP*, *EBF1*, *EZH2*, *HIST1H1B*, *HIST1H1C, HIST1H1D*, *HIST1H1E*, *KMT2C*, *KMT2D*, *MEF2B*, *PAX5*, *SMARCA4*, *TET2*), BCR-signaling (*BTK*, *CARD11*, *CD79B*, *CXCR4*, *FOXO1*, *IRF4*, *IRF8*, *KLF2*, *KLHL6*, *MS4A1*, *MYD88, PIK3CA*, *PIK3CD*, *PIK3R1*, *PLCG2*, *TNFAIP3*), immune response (*B2M*, *CD58*, *CTSS*, *IGLL5, TNFRSF14*), mTORC1 signaling (*ATP6AP1*, *ATP6AP2*, *ATP6V1B2*, *RRAGC*), and cell migration (*GNA13*, *GNAI2*).

NGS amplicon libraries were prepared from FFPE-extracted DNAs using a QIASeq targeted DNA custom panel (QIAGEN,) and sequenced on MiSeq or NextSeq platforms (Illumina, San Diego, CA, USA). Variants were classified according to the ACMG/AMP and AMP/ASCO/CAP [23,24] guidelines. Classification incorporated evidence from population and cancer databases (gnomAD v2.1.1, COSMIC, OncoKB, ClinVar, and Cancer Hotspots), in silico prediction tools (REVEL, BayesDel, MetaRNN, and SpliceAI), and published literature on FL. To establish the mutational profile and calculate the m7-FLIPI score, only variants classified as pathogenic or presumed pathogenic (P/PP) were considered.

### 2.6. Statistical Analysis

Statistical analyses were performed using IBM SPSS Statistics (version 23.0.0.0; Armonk, NY, USA) and R statistical software (v4.3.1) [25]). All tests were two-sided, with statistical significance set at *p*-value <0.05.

To compare TMB across patient groups, the Mann–Whitney U test was used for two groups, and the Kruskal–Wallis test was used for more than two groups, followed by Dunn’s post hoc test with Bonferroni correction when applicable. The Chi-squared test was used to compare TMB as a categorical variable across patient groups. The Wilcoxon signed-rank test compared TMB values at diagnosis and relapse.

Cox proportional hazards models were used to evaluate the impact of clinical and molecular factors on PFS, LSS, and OS. The proportional hazards assumption was confirmed for each variable, and statistical significance was determined by the likelihood ratio test. The model performance was evaluated using Harrell’s concordance index (C-index), Concordance Probability Estimate (CPE), Bayesian Information Criterion (BIC), and Akaike’s Information Criterion (AIC).

The maximally selected rank statistic, implemented in the R “maxstat” package (v0.7.25) [26]), was used to establish the optimal TMB cutoff point based on PFS, in order to stratify patients into high and low TMB groups.

## 3. Results

### 3.1. Patient Cohort

The clinical characteristics of the 135 patients are summarized in Table 1. The cohort is representative of the de novo FL and tFL populations described in previously reported series. In brief, the median age at diagnosis was 62 years in the de novo FL cohort and 59.5 years in tFL patients. At presentation, 87.4% of de novo FL patients presented with advanced-stage disease (Ann Arbor III–IV), and 29.4% had B-symptoms. In 56.6% of cases, bone marrow (BM) involvement was histologically proven. Among the 16 patients diagnosed with tFL, 62.5% presented with advanced-stage disease, 37.5% had B-symptoms, and 18.8% had bone marrow involvement.

The overall response rate in de novo FL patients was 95.8% (114/119), with 19.8% of patients (23/116) fulfilling the criteria of POD24. The median PFS was 107.9 months, with a 5-year PFS rate of 65.7% (95% CI: 56.8–76.0%), and no significant differences based on the treatment received (*p* = 0.870; Supplemental Figure S1), suggesting that treatment did not significantly influence disease progression in this cohort. Regarding tFL patients, the overall response rate was 81.2% (13/16), with 18.8% of patients experiencing progression within 2 years after receiving first-line therapy. Median PFS in this cohort was 120.4 months, with a 5-year PFS rate of 81.2% (95% CI: 64.2–100.0%).

### 3.2. Gene Mutational Landscape

No differences in the overall mutational distribution were observed between the de novo FL and tFL cohorts. All 135 patients had at least one SNV at diagnosis, with missense mutations being the most prevalent. SNVs were the most common variant type, with C > T transitions being the predominant form of base substitution. The median number of mutations per patient was 6 (range: 1–23), with an average of 5 missense and 1 nonsense mutations (Supplemental Figure S2A–E). Of the 409 genes analyzed, 236 were altered in at least one patient. The top ten most frequently mutated genes are presented in Supplemental Figure S2F.

A total of 853 different variants were identified at diagnosis, of which 57 were recurrent in more than one patient. The most recurring mutations were P/PP variants localized in mutational hotspots of 5 of the most frequently altered genes in FL, including: *CREBBP*, *EZH2*, *CARD11*, *BCL2*, and *FOXO1* (Supplemental Figure S3A). The most commonly mutated residue was Tyr646 in *EZH2*. Other frequently altered genes in FL, such as *KMT2D* and *TNFRSF14*, did not present alterations occurring in 3 or more patients (Supplemental Figure S3B).

### 3.3. Tumor Mutational Burden in De Novo FL Patients

**Figure 1:**
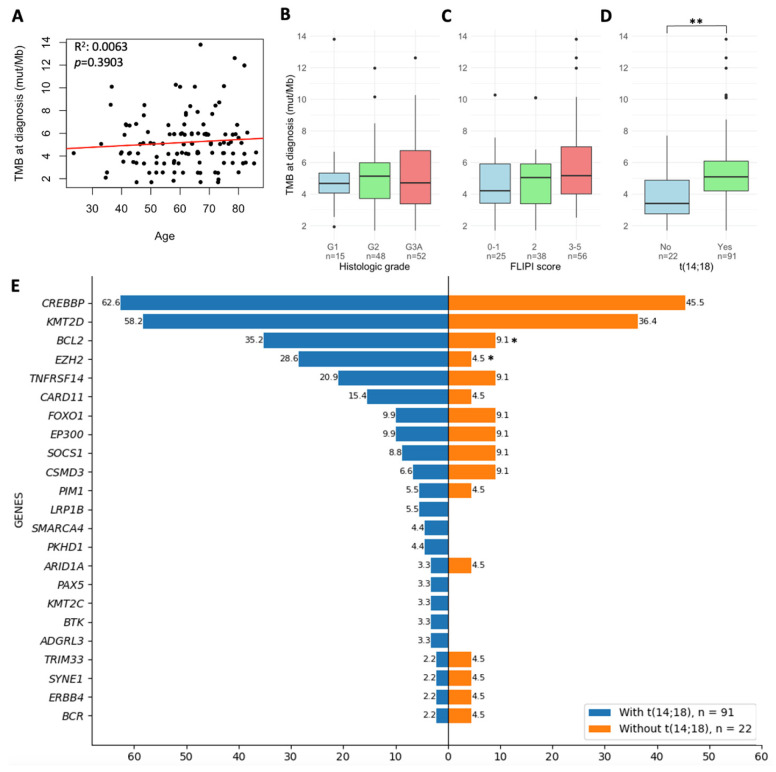
Tumor mutational burden (TMB) distribution in de novo follicular lymphoma (FL). (**A**) Scatter plot showing TMB values at diagnosis according to age at diagnosis, with a red trend line indicating the relationship between TMB and age. (**B-D**) Boxplots showing TMB values at diagnosis according to (**B**) histological grade, (**C**) Follicular Lymphoma International Prognostic Index (FLIPI) score, and (**D**) presence of the t(14;18) chromosomal translocation. (**E**) Histogram showing the mutational frequency of genes observed in at least three patients with or without the t(14;18). * *p*-value < 0.05, ** *p*-value < 0.01.

In the cohort of 119 patients with de novo FL, median TMB at diagnosis was 5.05 mut/Mb (range: 1.69–12.11 mut/Mb). No associations between TMB and clinical characteristics of the patients at diagnosis were observed, including age at diagnosis (*p* = 0.390), histological grades (*p* = 0.783), Ann Arbor stage (*p* = 0.610), ECOG performance status (*p* = 0.407), increased LDH (*p* = 0.219), increased β2-microglobulin (*p* = 0.886), FLIPI (*p* = 0.148), FLIPI-2 (*p* = 0.451), PRIMA-PI (*p* = 0.877), m7-FLIPI (*p* = 0.560), or FLPI24 (*p* = 0.144) (Figure 1A–C).

However, it was observed that patients harboring the t(14;18) chromosomal translocation presented with higher TMB values compared to t(14;18)-negative FL patients (median 5.09 vs. 3.40 mut/Mb, *p* = 0.004) (Figure 1D). Genetically, both groups of patients differed in the frequency of alterations in *BCL2* and *EZH2*, which were less frequent in t(14;18)-negative patients (Figure 1E).

### 3.4. TMB Association with the Mutational Profile

In the subset of 64 patients for whom additional targeted sequencing results on FL-relevant genes was available (described in Methods, Section 2.5), we explored the relationship between the mutational profile and TMB. A statistically significant positive correlation between TMB and the number of P/PP mutations in FL-relevant genes in these patients was observed (R = 0.3850, *p* = 0.002; Supplemental Figure S4). Furthermore, 98.4% of patients presented at least one P/PP mutation in a gene involved in epigenetic or transcriptional regulation. Moreover, a higher TMB was observed in patients harboring P/PP mutations in at least one gene involved in the cellular migration pathway (*GNA13* or *GNAI2*) (4.50 vs. 7.56 mut/Mb, *p* < 0.001). However, no association was found between TMB and the presence of P/PP mutations in other biological pathways (Supplemental Figure S5A).

At the individual gene level, P/PP mutations in the cellular migration pathway genes *GNA13* and *GNAI2* were independently associated with higher TMB (*p* = 0.010 and *p* = 0.004, respectively). Additionally, patients with P/PP mutations in *BCL2* or *TNFRSF14* exhibited higher TMB values, with a median TMB of 5.92 mut/Mb compared to 4.21 mut/Mb (*p* = 0.004), and 5.18 mut/Mb compared to 4.21 mut/Mb (*p* = 0.038), respectively. Higher TMB was also found to be associated with P/PP mutations in other genes, such as *TP53* or *BTK;* however, the limited representation of these mutations in our 64 FL cohort limits the interpretability of this finding (Supplemental Figure S5B).

### 3.5. Prognostic Value of TMB

For the de novo FL cohort, the optimal TMB threshold to stratify patients into low or high TMB groups was determined to be 2.55 mut/Mb. According to this threshold, 14.3% of the patients were classified as having low TMB (median 2.51 mut/Mb), and 85.7% as high TMB (5.14 ± 2.15 mut/Mb). The clinical characteristics were similar between the two patient groups (Table 2). However, the low-TMB group was enriched in patients with P/PP alterations in genes involved in the mTORC1 pathway (50.0% vs. 13.0%, *p* = 0.006), and none of the patients in this group presented alterations in genes involved in cellular migration (0.0% vs. 16.7%, *p* = 0.333) (Supplemental Figure S6). The low-TMB group also exhibited a trend towards a higher proportion of patients without the t(14;18) (35.3% vs. 16.7%, *p* = 0.074) (Table 2).

**Figure 2:**
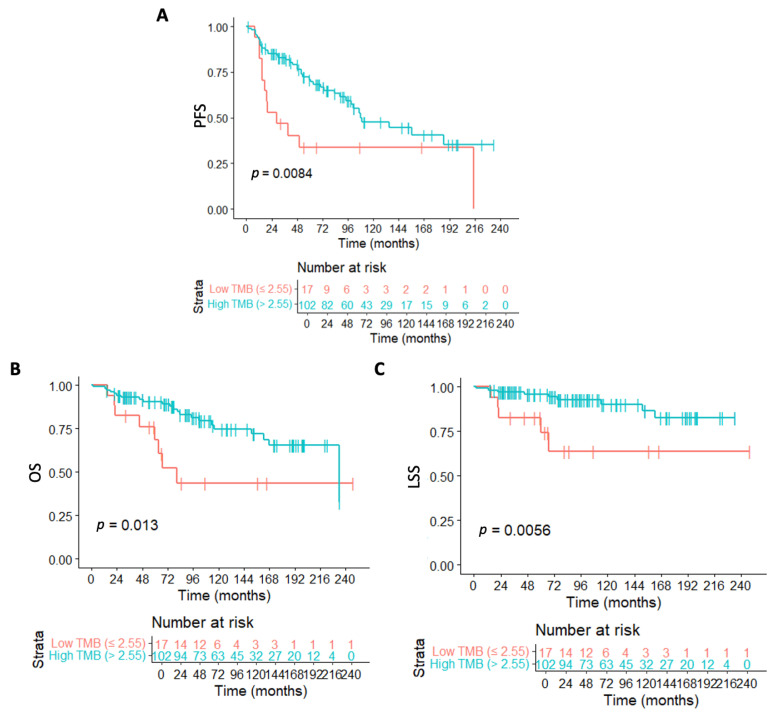
Impact of tumor mutational burden (TMB) on survival outcomes in de novo follicular lymphoma patients. (**A**) Progression-free survival (PFS), (**B**) overall survival (OS), and (**C**) lymphoma-specific survival (LSS) according to TMB levels (threshold: 2.55 mut/Mb). Light red indicates low TMB (≤2.55 mut/Mb), while light blue indicates high TMB (>2.55 mut/Mb).

PFS in the low-TMB patients was significantly shorter than in the high-TMB patients (*p* = 0.008). Five-year PFS was 33.6% (95%CI: 16.9–66.9%) and 71.0% (95%CI: 62.0–81.3%) for the low-TMB and high-TMB patients, respectively (Figure 2A). Differences in OS (*p* = 0.013) and LSS (*p* = 0.006) were also observed, with a median OS time of 80.8 months for low-TMB patients and 234.0 months for high-TMB patients (Figure 2B,C). Median LSS was not reached in either group.

POD24 was observed in 23 patients (19.3%), with a significantly higher incidence in the low-TMB group (47.1%) than in the high-TMB group (16.3%; *p* = 0.008).

TMB retained independent prognostic value for PFS, LSS, and OS in multivariate models that included FLIPI, and its addition consistently improved model performance across metrics such as C-index, CPE, BIC, and AIC (Supplemental Table S1). These results support the potential of TMB as a complementary biomarker in clinical risk stratification.

### 3.6. TMB Evaluation at Relapse

**Figure 3:**
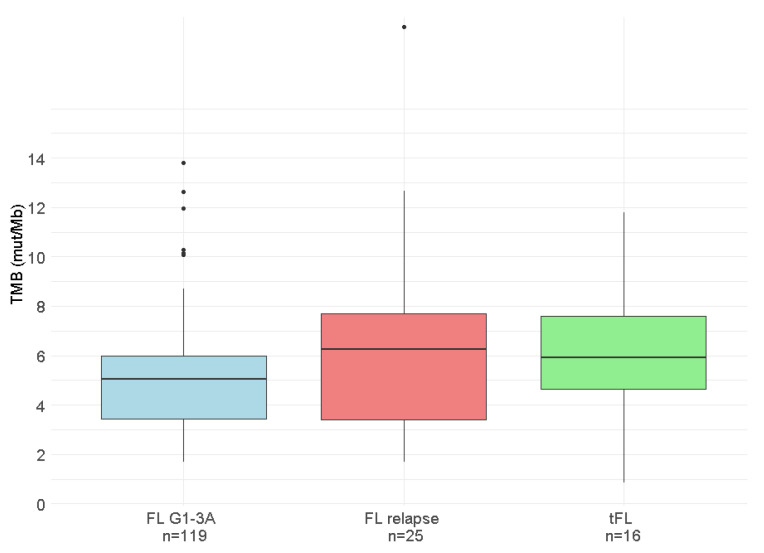
Tumor mutational burden (TMB) at diagnosis and relapse in de novo follicular lymphoma (FL) and at diagnosis in transformed follicular lymphoma (tFL) patients.

Among the 25 patients with available samples at the time of relapse, 8 biopsies showed transformation to DLBCL. Overall, no significant differences in TMB values were observed between the two time points (*p* = 0.427; Figure 3). Only one patient, which was POD24 and showed evidence of histological transformation to DLBCL at relapse, exhibited a significant increase in TMB at relapse (2.55 vs. 19.30 mut/Mb).

**Figure 4:**
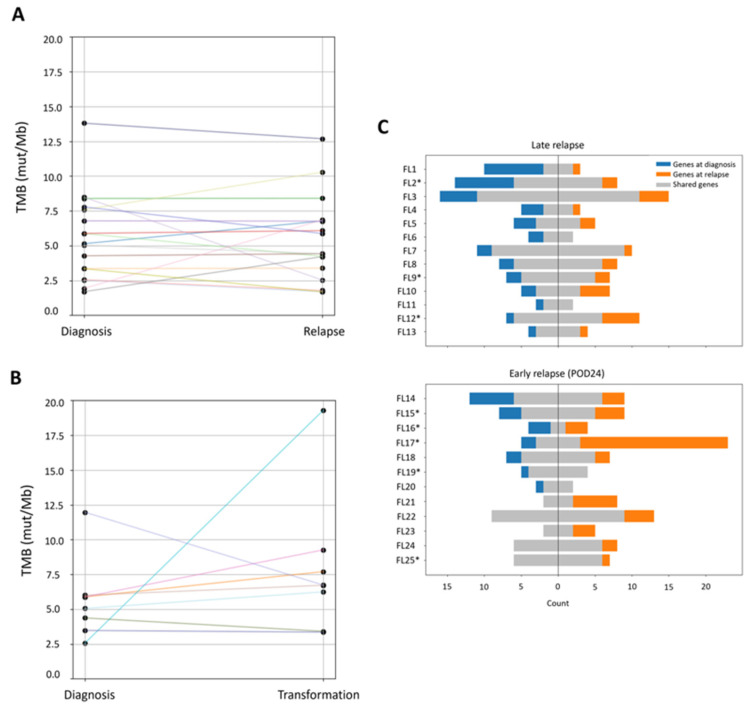
Tumor mutational burden (TMB) and genetic alterations in paired diagnosis and relapse/transformation samples. (**A**) TMB values of the 17 de novo follicular lymphoma (FL) patients who experienced relapse without histological transformation, and (**B**) of the 8 de novo FL patients with transformation to diffuse large B-cell lymphoma (DLBCL) at relapse. Colored lines connect the TMB values at diagnosis and relapse/transformation for each patient. (**C**) Number of alterations in paired diagnosis and relapse samples from patients who relapsed after (top panel) or before (bottom panel) 24 months of front-line treatment. Patients with transformation to DLBCL at relapse are marked with (*). On the left side of the graph, the total number of alterations identified at diagnosis is shown, with variants also present at relapse highlighted in grey and variants only identified at diagnosis in blue. On the right, alterations observed at relapse are presented, with grey indicating shared variants with the diagnostic sample and orange highlighting those exclusively found at relapse.

This finding raised the hypothesis that specific clinical subgroups might display distinct TMB dynamics over time. To explore this, patients were stratified according to transformation status, baseline TMB levels, presence of the t(14;18) translocation, FLIPI score, POD24, age, and type of treatment at diagnosis. However, none of these variables were associated with consistent TMB changes between diagnosis and relapse (Figure 4A,B).

In all cases, at least one common variant was identified between both samples, demonstrating a clonal relationship. In 42% of POD24 patients, all the variants present at the diagnostic sample were identified in the relapsed sample, suggesting the presence of an upfront aggressive resistant clone. In the remaining POD24 patients and in patients relapsing after 24 months, these clonal dynamics presented a different pattern because all patients showed at least one variant at diagnosis that became undetectable in the relapsed samples. However, 83.3% (10/12) of POD24 patients and 84.6% (11/13) of non-POD24 patients presented at relapse with at least one variant not identified at diagnosis (Figure 4C).

**Figure 5:**
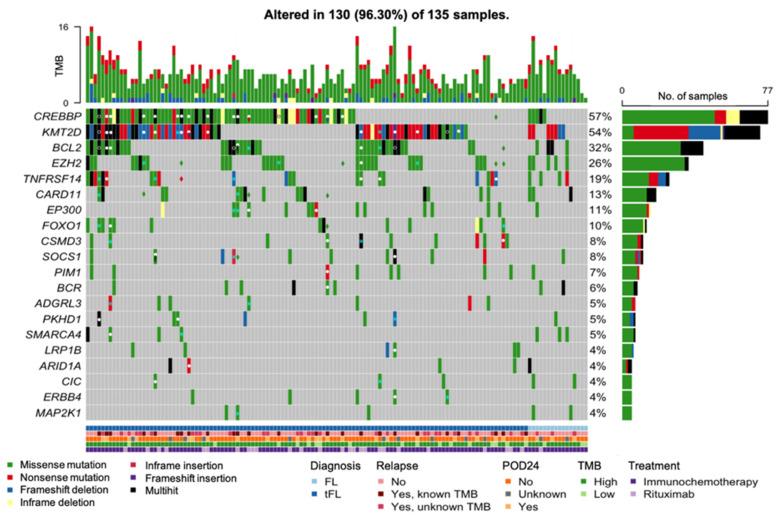
Oncoplot displaying the distribution of mutated genes in the 135 patients diagnosed with de novo FL or tFL. Mutations are color-coded according to mutation consequence: missense mutations (green), nonsense mutations (red), frameshift deletions (blue), inframe deletions (yellow), inframe insertion (dark red), frameshift insertion (purple), multihit (black).The total number of mutations identified in each patient, considering the 409 genes sequenced, is represented in the top row as an approximation to tumor mutational burden (TMB). In relapse samples, mutations present at both diagnosis and relapse are indicated by a white dot. In cases where a gene exhibits multiple alterations at diagnosis (multihit) and at least one, but not all, of these alterations are detected at the subsequent relapse, this is indicated by a white circle. Mutations identified only at diagnosis are marked with a light blue *, while mutations that emerge at relapse are represented with a diamond colored according to variant consequence. Patients are grouped according to their diagnosis, with those diagnosed with tFL shown on the right side of the graph. The bottom rows represent the diagnosis: de novo follicular lymphoma (FL; dark blue) and transformed follicular lymphoma (tFL; light blue); relapse status: patients without relapse (light red), relapsed patients of whom we sequenced the relapsed tumor sample to estimate TMB (medium red), relapsed patients without a sequenced relapse sample (dark red); the occurrence of relapse within 24 months of front-line therapy: no (dark orange), yes (light orange), 24-months follow-up not reached (grey); tumor mutational burden status (TMB): high (dark green), low (light green); treatment received: immunochemotherapy (dark purple), rituximab (light purple).

Despite the observed clonal evolution, the overall mutational frequency of individual genes at relapse was comparable to that at diagnosis (Supplemental Figure S7). This was due to the absence of recurrent losses or gains of mutations in specific genes. While some patients acquired a mutation in a particular gene at relapse, in others, a diagnostic mutation in that same gene became undetectable at relapse (Figure 5), resulting in a similar overall mutation frequency for that gene between the two time points. For instance, 4 patients acquired an *EZH2* mutation at relapse, whereas in 3 patients, the *EZH2* mutation became undetectable at relapse.

### 3.7. TMB in Patients Diagnosed with Transformed FL

**Figure 6:**
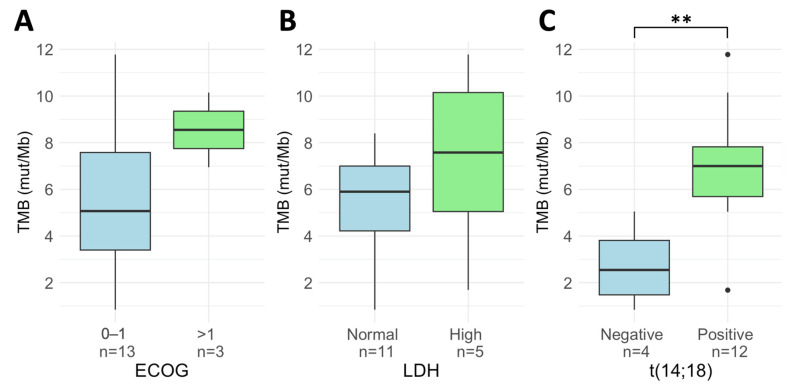
Tumor mutational burden (TMB) distribution in patients diagnosed with transformed follicular lymphoma (tFL). Boxplots show TMB values at diagnosis according to (**A**) Eastern cooperative oncology group (ECOG) performance status, (**B**) lactate dehydrogenase (LDH) levels, and (**C**) the presence of the t(14;18) chromosomal translocation. ** *p*-value < 0.01.

In the cohort of 16 tFL patients, the median TMB at diagnosis was 5.90 mut/Mb (range: 0.84–11.78 mut/Mb), which was comparable to that observed in the de novo FL cohort (*p* = 0.293; Figure 3). TMB was not associated with any clinical characteristic. However, as observed in the de novo FL patients, tFL t(14;18)-negative patients presented significantly lower TMB values compared to tFL t(14;18)-positive patients (2.54 vs. 7.00 mut/Mb, *p* = 0.013; Figure 6).

Regarding the prognostic value of TMB in this group of patients, the limited sample size prevented the determination of an optimal cutoff point for patient stratification, and conclusive survival analyses could not be performed. However, it is noteworthy that all three POD24 patients within this cohort presented TMB values above 2.55 mut/Mb.

## 4. Discussion

The prognosis of FL varies widely among patients, and the identification of prognostic biomarkers and the development of prognostic indices remain an unmet need in FL. In this study, we evaluated the prognostic relevance of TMB in FL patients receiving standard front-line treatment. From a biological perspective, the rationale for using TMB as a prognostic biomarker is based on the assumption that somatic mutations in coding regions may generate peptides with neoantigenic potential capable of eliciting T cell-mediated antitumor immune responses. Earlier studies attempted to directly quantify neoantigen load as a more precise measure of tumor immunogenicity; however, this approach requires complex computational prediction of antigen processing and HLA binding [27]. Consequently, TMB has emerged as a practical and reproducible surrogate of tumor neoantigenicity. Nevertheless, recent evidence indicates that some tumor neoantigens can arise from aberrant splicing events caused by intronic mutations or alterations in the splicing machinery [28]. These neoantigens would not be detected by DNA sequencing focused on exons and would require additional sequencing techniques such as mRNA sequencing together with more complex bioinformatic pipelines. Therefore, while TMB represents a clinically feasible and widely accepted approximation of a tumor’s immunogenic potential, it likely underestimates neoantigen generation. Future studies may help to understand the role of splicing-derived neoantigens in immune recognition and prognosis in FL. Currently, whole-exome sequencing continues to be the standard approach to determine TMB.

Although in our study TMB was estimated using a targeted NGS panel, instead of being determined by WES, panel-based TMB has shown a strong correlation with WES-derived TMB [18,20]. In particular, the commercial panel used in this study has been validated in solid tumors such as colorectal cancer, lung cancer, and melanoma [29,30]. While this panel has not been specifically validated in lymphomas, the available evidence supports the use of this panel-based TMB estimation for prognostic stratification.

TMB has been increasingly recognized as a potential prognostic biomarker in several types of cancer, including ovarian cancer [31], breast cancer [32], melanoma [33], and lung adenocarcinoma [34]. In line with these studies, our results show that de novo FL patients exhibiting low TMB values, at a cutoff value of 2.55 mut/Mb, demonstrated inferior PFS, LSS, and OS, independent of established prognostic factors such as FLIPI, histological grade, or disease stage. As TMB was derived from a ~1.2 Mb targeted panel, individual variants contribute discretely to the final TMB (approximately 0.8 mut/Mb), and TMB estimates close to the cutoff may be sensitive to small calling differences. To minimize this effect, filtering steps were optimized to ensure homogeneous variant calling across samples and discard potential artifacts. Overall, TMB distributions in the low- and high-TMB groups were separated from the cutoff, supporting the use of panel-based TMB and the established cutoff for patient stratification.

Studies addressing the relationship between TMB and prognosis in FL are limited and heterogeneous. Using WES, Tsukamoto et al. [35] reported a higher number of somatic mutations in high-risk FL patients; however, statistical significance was not maintained when only non-synonymous variants were considered. Patient risk stratification was based on the presence of peripheral blood or BM involvement, as this group of patients presented shorter PFS and OS. In contrast, we stratified patients according to TMB, and observed worse PFS and OS in patients with low TMB. Methodological differences may also contribute to the discrepant findings, as with WES, mutations associated with activation-induced cytidine deaminase (AID) were frequent and clustered in the immunoglobulin locus and *BCL2*, whereas our targeted panel did not include immunoglobulin regions. Mutations in these regions may reflect the ongoing somatic hypermutation rather than a generalized genomic instability that could have an impact on prognosis.

No additional experience has been reported so far in FL; however, controversial results have been shown for DLBCL, linking low TMB with poor outcomes but also with opposite results [36,37,38]. These discrepancies highlight the complexity of TMB as a prognostic factor and underscore the need for further research to better understand its role across different lymphoma subtypes and patient populations.

In our study, the association between higher TMB and improved clinical outcomes, including a lower likelihood of POD24, suggests that host antitumor immune activity may contribute to disease control in FL. Higher TMB is associated with increased neoantigen load, which may enhance T cell-mediated immune recognition and antitumor responses. Consistent with this rationale, TMB has been proposed as a predictive biomarker of response to immune checkpoint inhibitors (ICIs) across multiple cancer types [27,39,40]. However, in FL, the clinical activity of ICIs has been historically modest. Early studies of nivolumab monotherapy in the relapsed or refractory setting showed limited efficacy [41], and combinations such as pembrolizumab plus rituximab yielded higher but variable responses [42]. More recently, alternative strategies incorporating nivolumab priming followed by nivolumab plus rituximab in FL treatment naïve patients demonstrated encouraging results, with an overall response rate of 92% [43]. In this context, ICIs represent a biologically plausible therapeutic strategy for selected patients with FL. These findings support further investigation into the potential predictive role of TMB for ICI treatment in FL patients and raise the possibility that earlier introduction of ICIs, potentially through immune priming prior to cytotoxic therapy, may enhance clinical outcomes.

In our cohort, when considering TMB as a continuous variable, it was not associated with clinical characteristics, in line with findings from the Alliance A151303 study [44]. However, TMB was higher in t(14;18)-positive patients, potentially reflecting increased genomic instability driven by *BCL2* overexpression and impaired apoptosis. Similarly, patients harboring P/PP mutations in *BCL2* and *TNFRSF14* also presented with higher TMB values. *TNFRSF14*, which encodes a surface receptor involved in immune response regulation and Fas-induced apoptosis [45], has also been associated with high TMB in other tumor types [46]. Altogether, these results suggest that genetic alterations leading to apoptosis inhibition may contribute to the accumulation of somatic mutations in FL.

The mutational profile of our de novo FL cohort, including frequent alterations in *CREBBP*, *KMT2D*, *BCL2*, and *EZH2*, is consistent with previous FL studies [47,48], supporting the reliability of our sequencing approach and confirming that our cohort reflects the characteristic genetic landscape of FL.

Inactivating mutations in genes involved in DNA repair mechanisms have also been linked to higher TMB [49]. However, only 2.5% of patients in our cohort exhibited *TP53* mutations; therefore, no definitive conclusions could be drawn. Nevertheless, the three patients harboring *TP53* mutations were among those with the highest TMB values. We observed an association between TMB levels above 2.55 mut/Mb and the presence of P/PP mutations in *GNA13* and *GNAI2*. These two G protein subunits are involved in several transmembrane signaling pathways, including cell migration and invasion.

When considering TMB as a discrete variable, the high-TMB group was enriched not only in t(14;18) and *GNA13/GNAI2* mutations, as expected, but also presented a lower frequency of P/PP mutations in genes related to the mTOR pathway. Despite the evidence in the existing literature suggesting a link between mTOR signaling and high TMB due to its association with DNA damage repair pathways and microsatellite instability [50,51], it is important to note that these discrepancies may arise from differences in the genes analyzed, given the limited overlap in gene inclusion across studies.

To further explore TMB dynamics, the analysis was extended to include relapse samples. The median TMB remained stable over time, with only minor fluctuations across patients, and no association was found between the TMB changes and clinical characteristics such as POD24, t(14;18) translocation, or type of treatment. Despite the evolution of the mutational landscape in all patients, no specific gene consistently gained or lost mutations at relapse, suggesting a relatively stable mutational frequency for most genes. We did observe that in 42% of POD24 patients, all the mutations detected at diagnosis were also detected at relapse, while in all non-POD24 patients, at least one mutation became undetectable. In the context of personalized medicine, our findings highlight the importance of reassessing the mutational status of selected genes at relapse, especially since mutations relevant to targeted therapies may emerge or disappear over time. In this regard, in our cohort, 4 patients acquired an *EZH2* mutation at relapse, which is clinically actionable and targeted by Tazemetostat, while in 3 other patients, a mutation in that gene became undetectable. Previous studies have reported increased mutation frequencies in specific genes at relapse, including *TP53*, *STAT6*, and *MYD88* [52]. We did not detect the acquisition of mutations in these genes in our cohort. However, the mutation frequency of these genes is relatively low, even in relapsed FL, which limits the statistical power to detect significant changes within our relatively small cohort of 25 relapsed patients.

Finally, TMB has been reported to increase at the time of histological transformation [53,54]. Patients in our tFL cohort showed a similar median TMB compared to the de novo FL cohort. This discrepancy may be explained by the differing clinical contexts, as prior studies have focused on sequentially transformed lymphomas after FL therapy, whereas our analysis was restricted to treatment-naïve diagnostic samples of tFL. Although the limited number of tFL cases included in the study restricted the evaluation of the prognostic value of TMB within this subgroup, high TMB in tFL remained associated with the presence of the t(14;18) chromosomal translocation.

This study has some limitations. Despite the relatively large overall cohort, analyses concerning patients with tFL at diagnosis were limited by small sample sizes, reducing statistical power and precluding more detailed conclusions. Additionally, TMB was estimated using targeted sequencing, which, although clinically practical, may not capture the full spectrum of mutations compared to whole-exome or whole-genome sequencing. These limitations are mitigated by the extensive clinical data and long-term follow-up in this cohort; however, future prospective studies with larger and more uniform cohorts will be needed to validate these findings and assess their clinical utility.

## 5. Conclusions

In summary, patients with high TMB presented longer PFS, LSS, and OS. Our findings support the potential of TMB as a prognostic biomarker in FL, offering insights beyond established clinical indices.

## Figures and Tables

**Table 1:** Patients’ clinical characteristics at diagnosis.

	FL 1-3A (*n* = 119)	Transformed FL (*n* = 16)
Age at diagnosis, median in years (range)	62 (24–86)	59.5 (40–81)
Gender		
Male	62 (52.1%)	12 (75.0%)
Female	57 (47.9%)	4 (25.5%)
Histological grade ^1^		
1–2	63 (54.8%)	
3A	52 (45.2%)	
t(14;18) ^2^		
No	22 (19.5%)	4 (25.0%)
Yes	91 (80.5%)	12 (75.0%)
Ki67 index ^2^		
≤30%	81 (71.7%)	0
>30%	32 (28.3%)	16 (100%)
Ann Arbor stage		
I/II	15 (12.6%)	6 (37.5%)
III/IV	104 (87.4%)	10 (62.5%)
B-Symptoms		
No	84 (70.6%)	10 (62.5%)
Yes	35 (29.4%)	6 (37.5%)
ECOG ^1^		
0–1	103 (89.6%)	14 (87.5%)
2–4	12 (10.4%)	2 (12.5%)
BM involvement ^3^		
No	49 (43.4%)	12 (80.0%)
Yes	64 (56.6%)	3 (20.0%)
LDH		
Normal	95 (79.8%)	11 (68.75%)
High	24 (20.2%)	5 (31.25%)
β2-microglobulin^4^		
Normal	79 (66.4%)	15 (100%)
High	40 (33.6%)	0
FLIPI score		
Low (0–1)	25 (21.0%)	5 (31.25%)
Intermediate (2)	38 (31.9%)	5 (31.25%)
High (3–5)	56 (47.1%)	6 (37.5%)
FLIPI-2 score ^1^		
Low (0)	14 (12.1%)	5 (33.3%)
Intermediate (1–2)	55 (47.4%)	9 (60.0%)
High (3–5)	47 (40.5%)	1 (6.7%)
m7FLIPI score ^5^		
Low (<0.8)	46 (71.9%)	
High (>0.8)	18 (28.1%)	
Induction regimen		
R/O-CHOP like	66 (55.5%)	15 (93.75%)
R-CVP	16 (13.4%)	0
R/O-B	7 (5.9%)	0
R monotherapy	30 (25.2%)	1 (6.25%)

^1^ Information not available in 4 cases. ^2^ Information not available in 6 cases. ^3^ Information not available in 7 cases. ^4^ Information not available in 1 case. ^5^ Information not available in 71 cases. ECOG: Eastern cooperative oncology group, BM: Bone marrow, LDH: Lactate dehydrogenase, FLIPI: Follicular lymphoma international prognostic index, R/O-CHOP: Rituximab/obinutuzumab, cyclophosphamide, doxorubicin hydrochloride, vincristine, prednisone, R-CVP: Rituximab, cyclophosphamide, vincristine, and prednisone, R/O-B: Rituximab/obinutuzumab and bendamustine, R: Rituximab.

**Table 2:** Clinical features of de novo FL patients stratified according to TMB.

	Low-TMB (≤2.55 mut/Mb) (*n* = 17)	High-TMB (>2.55 mut/Mb) (*n* = 102)	*p*-Value
Age at diagnosis, median in years (range)	58 (34–73)	63 (24–86)	0.256
Gender			
Male	10 (58.8%)	52 (51.0%)	0.549
Female	7 (41.2%)	50 (49.0%)	
Histological grade ^1^			
1–2	8 (50%)	55 (55.6%)	0.679
3A	8 (50%)	44 (44.4%)	
t(14;18) ^2^			
No	6 (35.29%)	16 (16.67%)	0.074
Yes	11 (64.71%)	80 (83.33%)	
Ki67 index ^2^			
≤30%	13 (76.5%)	68 (70.8%)	0.854
>30%	4 (23.5%)	28 (29.2%)	
Ann Arbor stage			
I/II	2 (11.8%)	13 (12.7%)	1.000
III/IV	15 (88.2%)	89 (87.3%)	
B-Symptoms			
No	11 (64.7%)	73 (71.6%)	0.565
Yes	6 (35.3%)	29 (28.4%)	
ECOG ^1^			
0–1	17 (100%)	86 (87.8%)	0.209
2–4	0 (0%)	12 (12.2%)	
BM involvement ^2^			
No	7 (41.2%)	42 (43.7%)	0.844
Yes	10 (58.8%)	54 (56.3%)	
LDH			
Normal	12 (70.6%)	83 (81.4%)	0.305
High	5 (29.4%)	19 (18.6%)	
β2-microglobulin			
Normal	13 (76.5%)	66 (64.7%)	0.416
High	4 (23.5%)	36 (35.3%)	
FLIPI score			
Low (0–1)	4 (23.5%)	21 (20.6%)	0.951
Intermediate (2)	5 (29.4%)	33 (32.3%)	
High (3–5)	8 (47.1%)	48 (47.1%)	
FLIPI-2 score ^3^			
Low (0)	1 (5.9%)	13 (13.1%)	0.519
Intermediate (1–2)	10 (58.8%)	45 (45.5%)	
High (3–5)	6 (35.3%)	41 (41.4%))	
m7-FLIPI score ^4^			
Low (<0.8)	7 (70.0%)	39 (72.2%)	1.000
High (>0.8)	3 (30.0%)	15 (27.8%)	
Induction regimen			
R/O-CHOP	9 (52.94%)	57 (55.88%)	0.977
R-CVP	2 (11.77%)	14 (13.73%)	
R/O-B	1 (5.88%)	6 (5.88%)	
R monotherapy	5 (29.41%)	25 (24.51%)	

^1^ Information not available in 4 cases. ^2^ Information not available in 6 cases. ^3^ Information not available in 3 cases. ^4^ Information not available in 55 cases. TMB: Tumor mutational burden, ECOG: Eastern cooperative oncology group, BM: Bone marrow, LDH: Lactate dehydrogenase, FLIPI: Follicular lymphoma international prognostic index. R/O-CHOP: Rituximab/obinutuzumab, cyclophosphamide, doxorubicin hydrochloride, vincristine, prednisone, R-CVP: Rituximab, cyclophosphamide, vincristine, and prednisone, R/O-B: Rituximab/obinutuzumab and bendamustine, R: Rituximab.

## Data Availability

The DNA sequencing data from follicular lymphoma patients have been deposited at the EGA database under accession number EGAD50000002087.

## Supplementary Materials

The following supporting information can be downloaded at: https://www.mdpi.com/article/10.3390/cancers18050737/s1. Table S1: Prognostic value and predictive performance of tumor mutational burden (TMB) in follicular lymphoma; Figure S1: Progression-free survival (PFS) according to the first-line treatment regimen in de novo follicular lymphoma patients; Figure S2: Variant distribution summary in FL and tFL tumors at diagnosis; Figure S3: Genetic variants encountered in the most commonly mutated genes; Figure S4: Correlation between the number of pathogenic or presumed pathogenic (P/PP) mutations within the 64 FL-related genes and tumor mutational burden (TMB) in 64 FL patients; Figure S5: Association of tumor mutational burden (TMB) with the genetic landscape; Figure S6: Distribution of patients according to tumor mutational burden (TMB) values (cutoff point of 2.55mut/Mb) and the presence of genetic alterations in biological pathways; Figure S7: Frequency of gene mutations in paired diagnosis and progression samples.
