# Supplementary material for: Tumor Mutational Burden as a Prognostic Biomarker in Follicular Lymphoma
Source: Cancers (Basel). 2026 Feb 25;18(5):737. doi: 10.3390/cancers18050737 (PMC12985221; doi:10.3390/cancers18050737)
Supplement: Supplementary file 1 [file cancers-18-00737-s001.zip › cancers-4146371-supplementary.pdf]

Supplemental Data

Evaluation of the role of tumor mutational burden as a prognostic biomarker in follicular lymphoma

Lafuente M. *et al*.

S1. Supplemental Tables 2

S2. Supplemental Figures 3

S1. Supplemental tables

Table S1. Prognostic value and predictive performance of tumor mutational burden (TMB) in follicular lymphoma. (A) Predictive performance of Follicular Lymphoma International Prognostic Index (FLIPI), TMB, and their combination for progression-free survival (PFS), lymphoma-specific survival (LSS), and overall survival (OS), measured by C-index, concordance probability estimate (CPE), Bayesian Information Criterion (BIC), and Akaike Information Criterion (AIC). (B) Multivariate Cox regression analysis for PFS, LSS, and OS showing hazard ratios (HRs) and 95% confidence intervals for FLIPI score (3–5) and high TMB (>2.55 mut/Mb).

(A) Predictive performance metrics

	**PFS**				**LSS**				**OS**			
	C-Index	CPE	BIC	AIC	C-Index	CPE	BIC	AIC	C-Index	CPE	BIC	AIC
FLIPI score (3–5)	0.632	0.611	418.760	618.373	0.654	0.628	118.766	216.068	0.654	0.639	240.854	411.070
TMB >2.55	0.580	0.550	424.424	624.835	0.649	0.576	117.482	216.337	0.598	0.558	246.513	417.716
FLIPI score (3–5) + TMB >2.55	0.657<br>p = 0.002**	0.657	413.186	613.761	0.719<br>p = 0.012*	0.680	115.050	213.520	0.703<br>p = 0.008**	0.677	237.284	408.718

(B) Multivariate Cox regression

	**PFS**		**LSS**		**OS**	
	HR (95%CI)	p-value	HR (95%CI)	p-value	HR (95%CI)	p-value
FLIPI score (3–5)<br>+ TMB >2.55	3.113 (1.722–5.627)<br>0.304 (0.153–0.604)	<0.001 ***<br><0.001 ***	3.554 (1.100–11.484)<br>0.203 (0.066–0.625)	0.034 *<br>0.005 **	4.017 (1.759–9.177)<br>0.288 (0.125–0.666)	<0.001 ***<br>0.004 **

S2. Supplemental figures

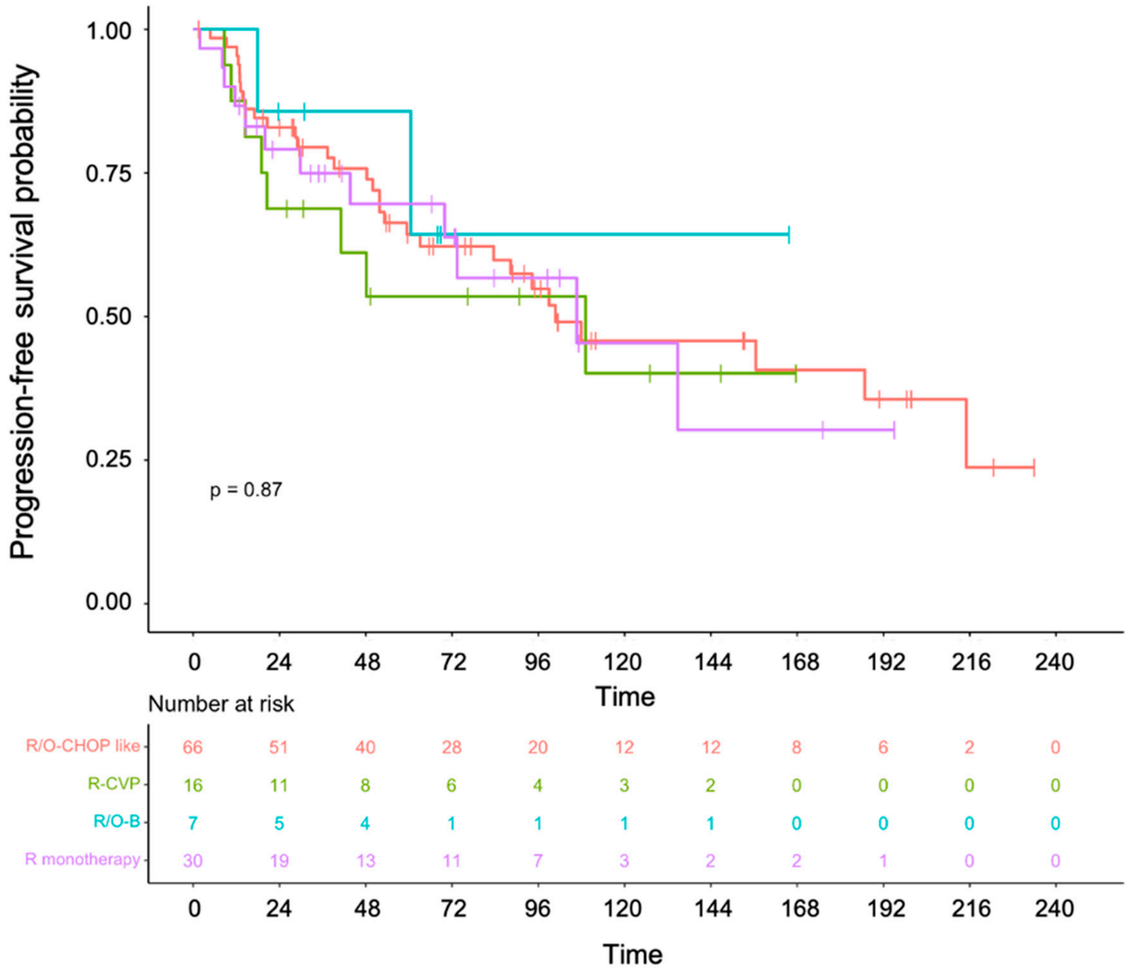

Figure S1. Progression-free survival (PFS) according to the first-line treatment regimen in de novo follicular lymphoma patients. The number of patients at risk is shown below the plot.

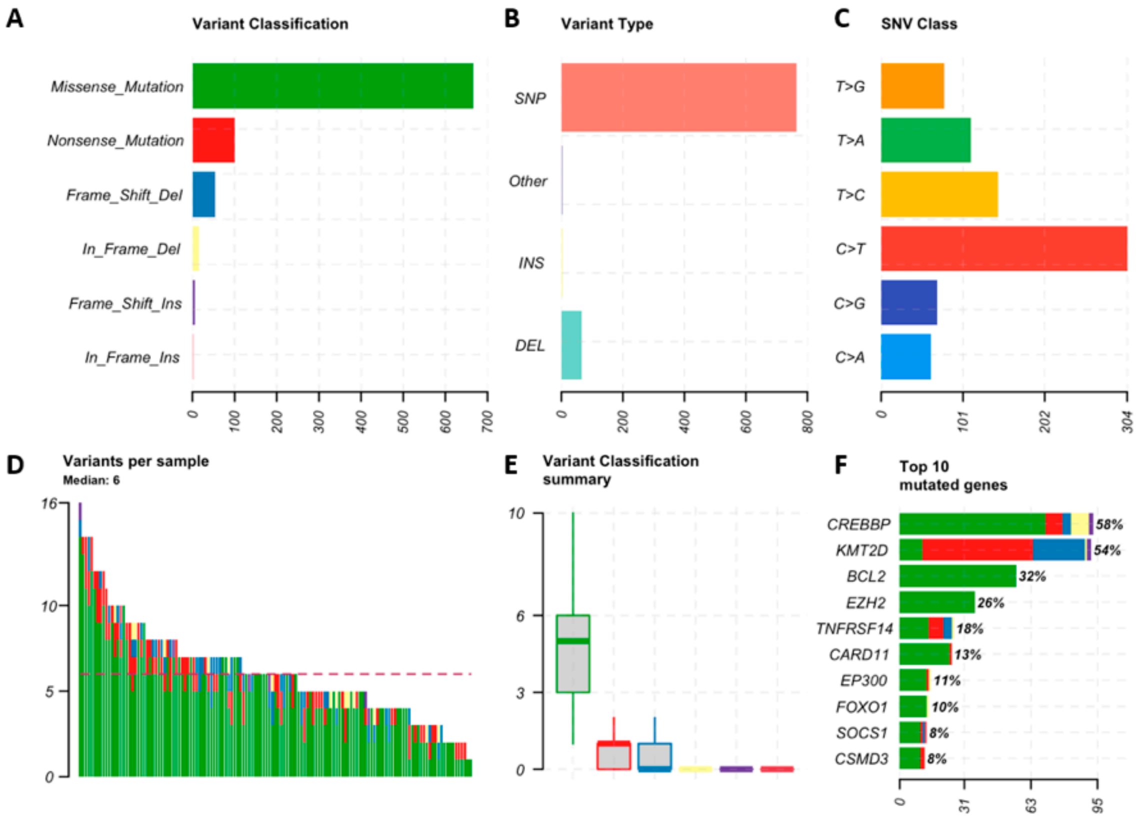

Figure S2. Variant distribution summary in FL and tFL tumors at diagnosis. Variant count according to (A) the functional effect, (B) variant types (SNP: single nucleotide polymorphism; Other: start site loss; INS: insertion; DEL: deletion), and (C) class of base substitution. (D) Number of mutations and distribution per sample. The dashed line represents the median value of 6 mutations/patient. (E) Variant classification summary per sample. (F) Top 10 mutated gene counts and frequency of patients harboring a mutation.

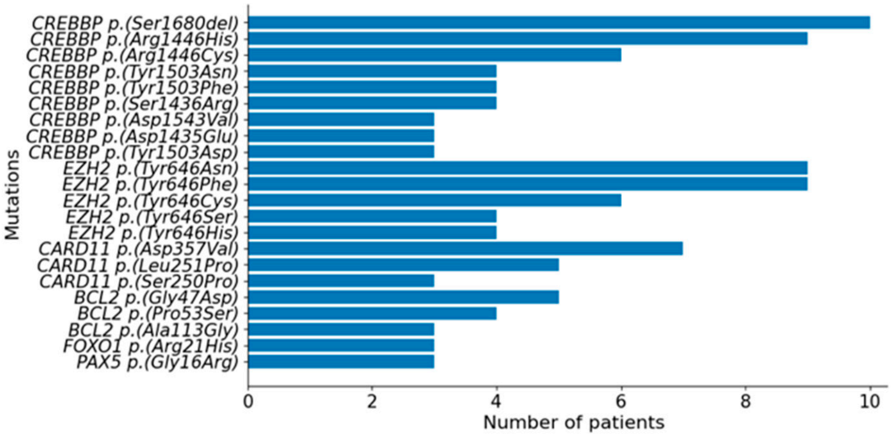

(a)

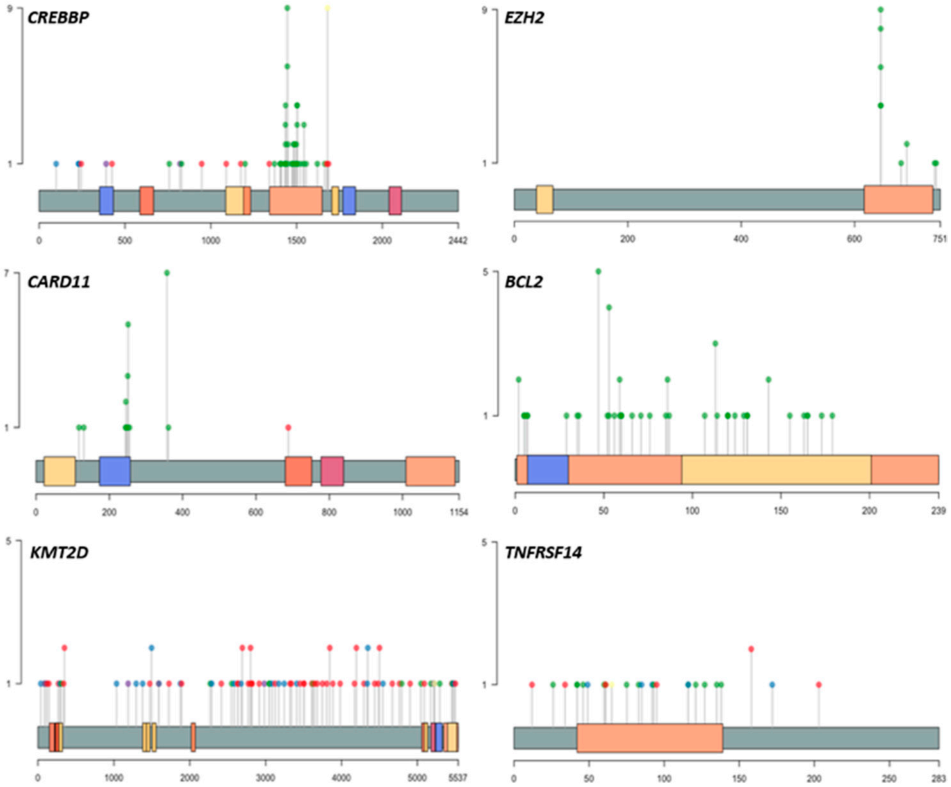

(b)

Figure S3. Genetic variants encountered in the most commonly mutated genes. (A) Recurrent mutations detected in at least three FL or tFL patients, showing the number of cases per mutation. (B) Lollipop plots representing all the mutations encountered across the 135 patients in the 6 most recurrently altered genes in our cohort. Missense variants are represented in green, nonsense variants in red, frameshift deletions in blue, in-frame deletions in yellow, and frameshift insertions in purple.

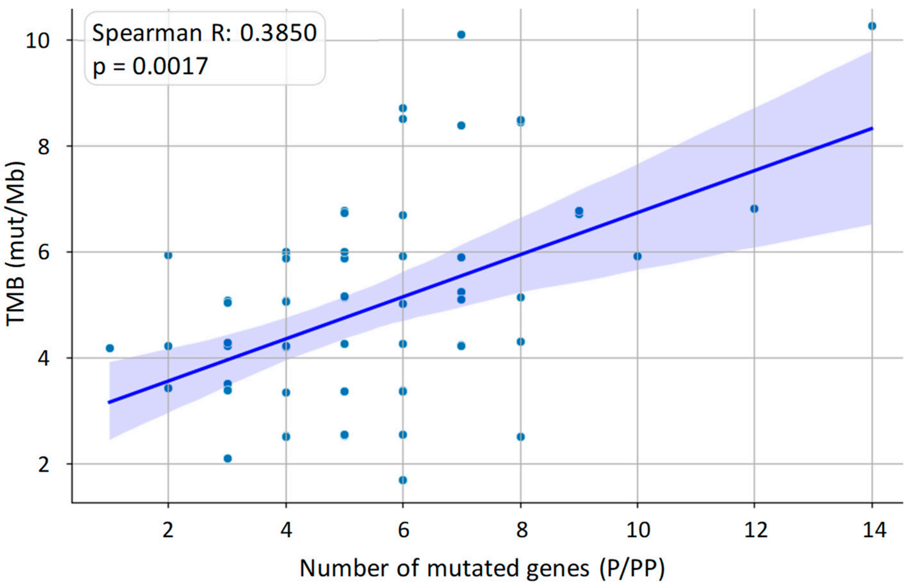

Figure S4. Correlation between the number of pathogenic or presumed pathogenic (P/PP) mutations within the 64 FL-related genes and tumor mutational burden (TMB) in 64 FL patients.

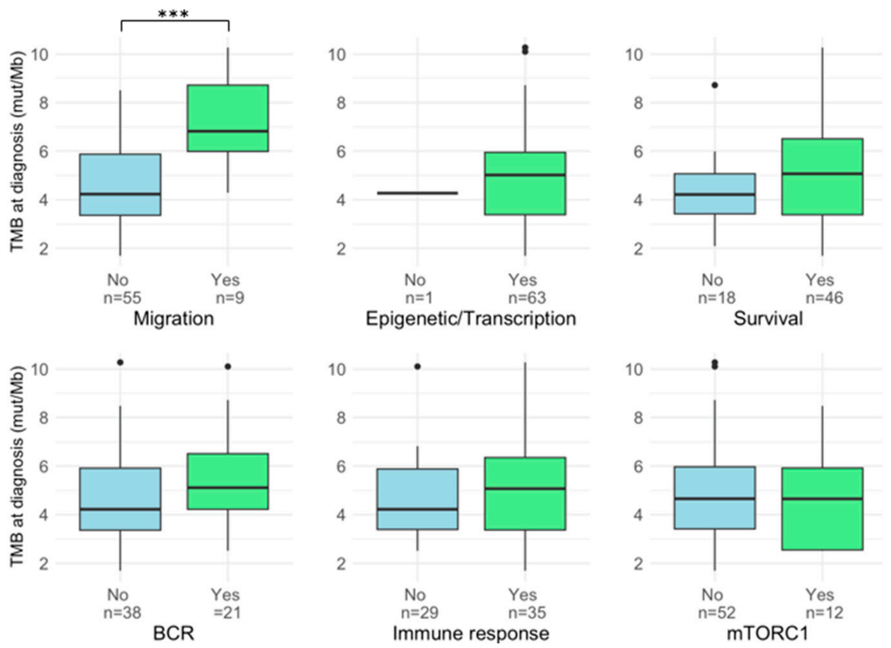

(a)

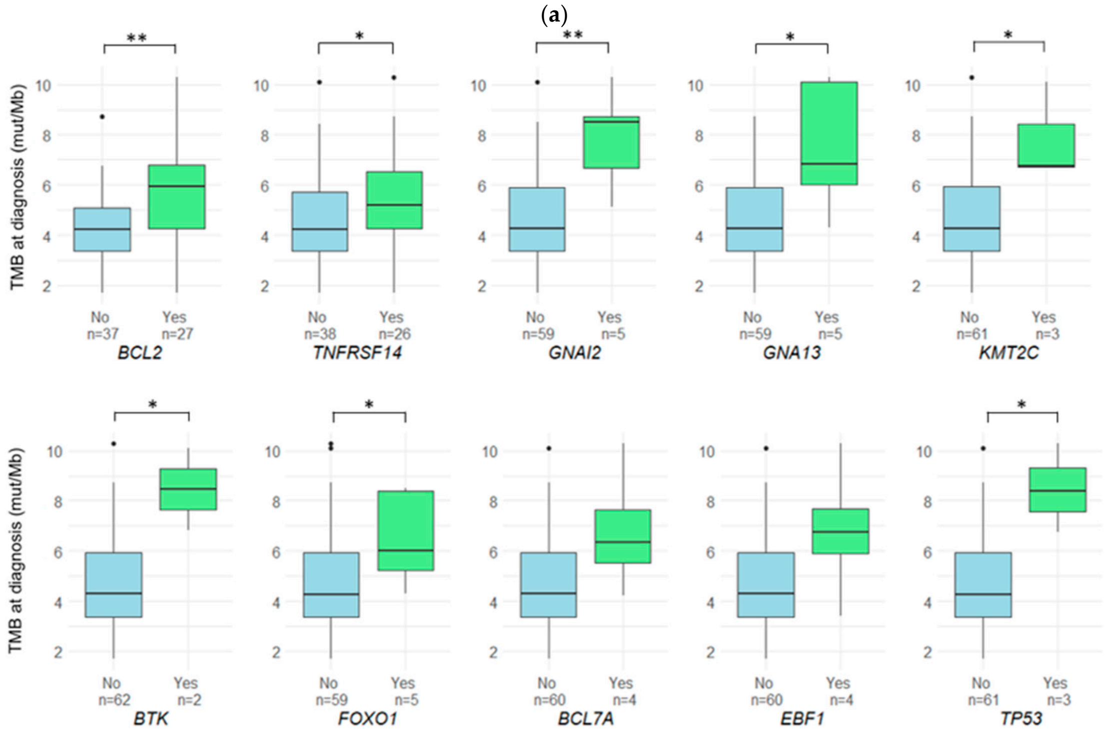

(b)

Figure S5. Association of tumor mutational burden (TMB) with the genetic landscape. TMB in follicular lymphoma patients grouped by (A) the presence of pathogenic or presumed pathogenic (P/PP) mutations in genes associated with six different biological pathways, and (B) the presence of P/PP mutations in 10 genes frequently altered in follicular lymphoma. *p < 0.05, **p < 0.01, ***p < 0.001.

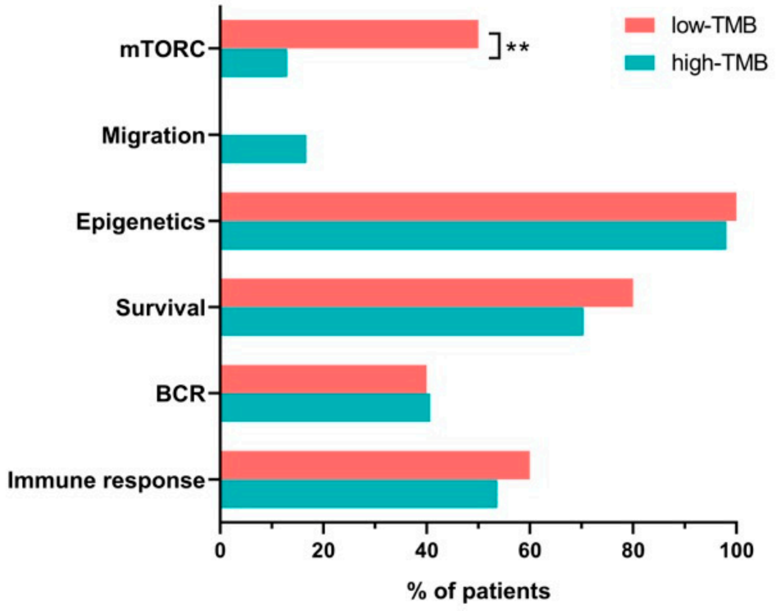

Figure S6. Distribution of patients according to tumor mutational burden (TMB) values (cutoff point of 2.55mut/Mb) and the presence of genetic alterations in biological pathways. ** p-value < 0.01.

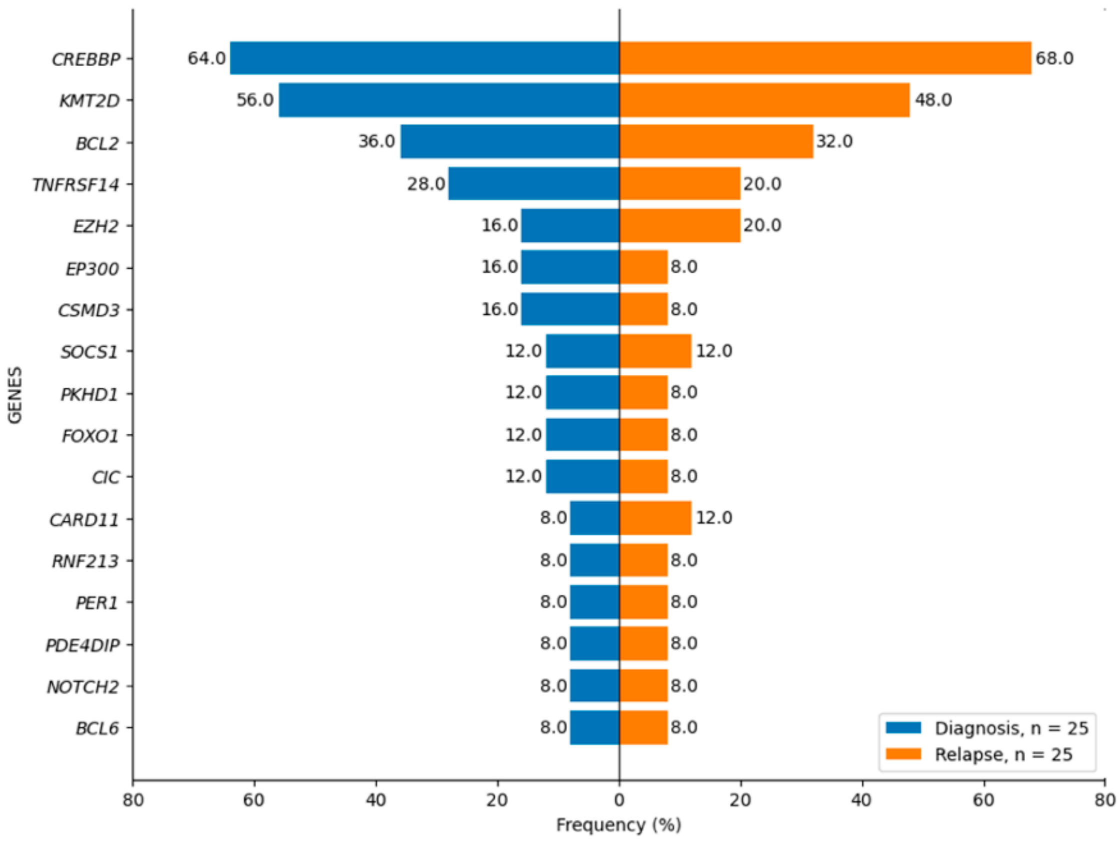

Figure S7. Frequency of gene mutations in paired diagnosis and progression samples. Histogram showing the frequency of mutations in distinct genes in 25 follicular lymphoma patients. Mutations identified at diagnosis are represented on the left side of the graph (blue), while those identified at progression are shown on the right (orange).
